# Impact of the EILA application on smoking cessation among hospital workers: a randomised controlled trial

**DOI:** 10.1136/oemed-2025-110157

**Published:** 2026-06-08

**Authors:** Erik Schneider, Wiebke Wanner, Mareike Lüthgen, Dominik Edward Pawlak, Henrike Alexandra Faesser, Kai Mortensen, Daniel Drömann, Tobias Jagomast, Klaas F Franzen

**Affiliations:** 1 Pulmonology (Department of Medicine III), University Hospital Schleswig-Holstein, Lübeck, Germany; 2 Cardiology (Department of Medicine II), University Hospital Schleswig-Holstein, Lübeck, Germany; 3 Herzzentrum Kiel, Kiel, Germany; 4 German Center for Lung Research, Giessen, Germany

**Keywords:** Occupational Health, Smoking, Health Personnel

## Abstract

**Objectives:**

Smoking remains a major preventable cause of lung and cardiovascular disease. This randomised controlled trial evaluated EILA, a mobile application using artificial intelligence, for promoting smoking cessation among hospital workers.

**Methods:**

A randomised controlled trial was conducted. Hospital workers (n=43) were recruited from a German university hospital from May 2022 to March 2023. Inclusion criteria comprised age ≥18, Fagerström score ≥3 or 10 cigarettes per day, ownership of a suitable smartphone, motivation to quit smoking and no addiction to other psychoactive substances. Participants were randomised by sealed envelopes to EILA intervention (n=25) or a wait-list control (n=18) receiving a primary care-style intervention, with crossover after 3 months. The primary outcome was the cessation rate. Secondary outcomes comprised nicotine dependence, mental health and cardiovascular risk, all measured using validated questionnaires.

**Results:**

At the 6-month follow-up, the relative risk for continued smoking was reduced to 0.91 (n=51, 95% CI 0.7 to 1.2), and nicotine dependence, measured by the Fagerström Test, decreased significantly from 4.41±1.93 to 3.32±2.69 (n=19, p=0.014). PROCAM score for cardiovascular risk was reduced from 35.22±12.47 to 31.9±13.24 (n=36, p=0.008).

**Conclusion:**

The digital health application EILA was linked to increased smoking cessation rates, although without achieving statistical significance. Nevertheless, the results indicate reductions in nicotine dependence and cardiovascular risk, supporting EILA as a scalable and flexible intervention for promoting health in hospital workers.

**WHAT IS ALREADY KNOWN ON THIS TOPIC**

- Digital health applications for smoking cessation are gaining importance as resource-efficient alternatives to traditional programmes. However, only a few have obtained regulatory approval as licensed health products, and evidence on their efficacy remains limited.

**WHAT THIS STUDY ADDS**

- This study examines the use of a mobile smoking cessation application among hospital workers, a population facing unique challenges in attending conventional programmes due to irregular working hours and high stress levels that often trigger smoking.

**HOW THIS STUDY MIGHT AFFECT RESEARCH, PRACTICE OR POLICY**

- Digital smoking cessation applications could become standard tools for healthcare workers, supporting smoking reduction or cessation. This may not only improve workers’ health but also enhance healthcare delivery by reducing smoking-related morbidity within the workforce.

## Introduction

 Cigarette smoking is a major risk factor for cardiovascular disease and lung cancer, imposing substantial healthcare costs in Germany, estimated at €90,000 to €530,000 per smoker.^1 2^ The rising prevalence of smoking among young adults in Germany underscores the urgent need for effective smoking cessation strategies.^3^ Hospital workers represent a subgroup of particular interest, as this population is well known to have an increased risk of developing nicotine dependence.^4^ Smoking cessation is challenged by nicotine addiction and various triggers associated with smoking behaviour. Owing to their irregular work schedules, hospital workers rarely have access to conventional tobacco cessation programmes, highlighting the urgent need for flexible, alternative interventions.^5^

Smart interventions have gained attention for their accessibility and potential to support smoking cessation, especially with the recent authorisation of digital health applications by the German Federal Institute for Drugs and Medical Devices.^6–9^ Furthermore, Article 14 of the WHO Framework Convention on Tobacco Control emphasises the importance of accessible and affordable interventions to support individuals in quitting tobacco.^10^ This study aims to address the gap in targeted smoking cessation interventions for high-risk populations, such as hospital workers.^11^

The first digital health applications approved by the German Federal Institute for Drugs and Medical Devices for smoking cessation are the ‘NichtraucherHelden-App’ and ‘Smoke Free’.^12–14^ These approved applications represent a significant advance in using digital tools for tobacco addiction treatment, highlighting the potential of smartphone-based interventions as accessible, on-demand support for smoking cessation. They are grounded in principles of cognitive-behavioural psychology.^15 16^

In contrast, the EILA application integrates behavioural psychology principles with artificial intelligence to deliver personalised smoking cessation support. This study evaluated the impact of this application on smoking cessation rates among hospital workers with nicotine addiction in comparison to a guideline-based primary care-style smoking cessation consultation.^17^ Secondary outcomes included changes in nicotine dependence, mental health and cardiovascular risk.

## Material and methods

### Study design and population

**Figure 1:**
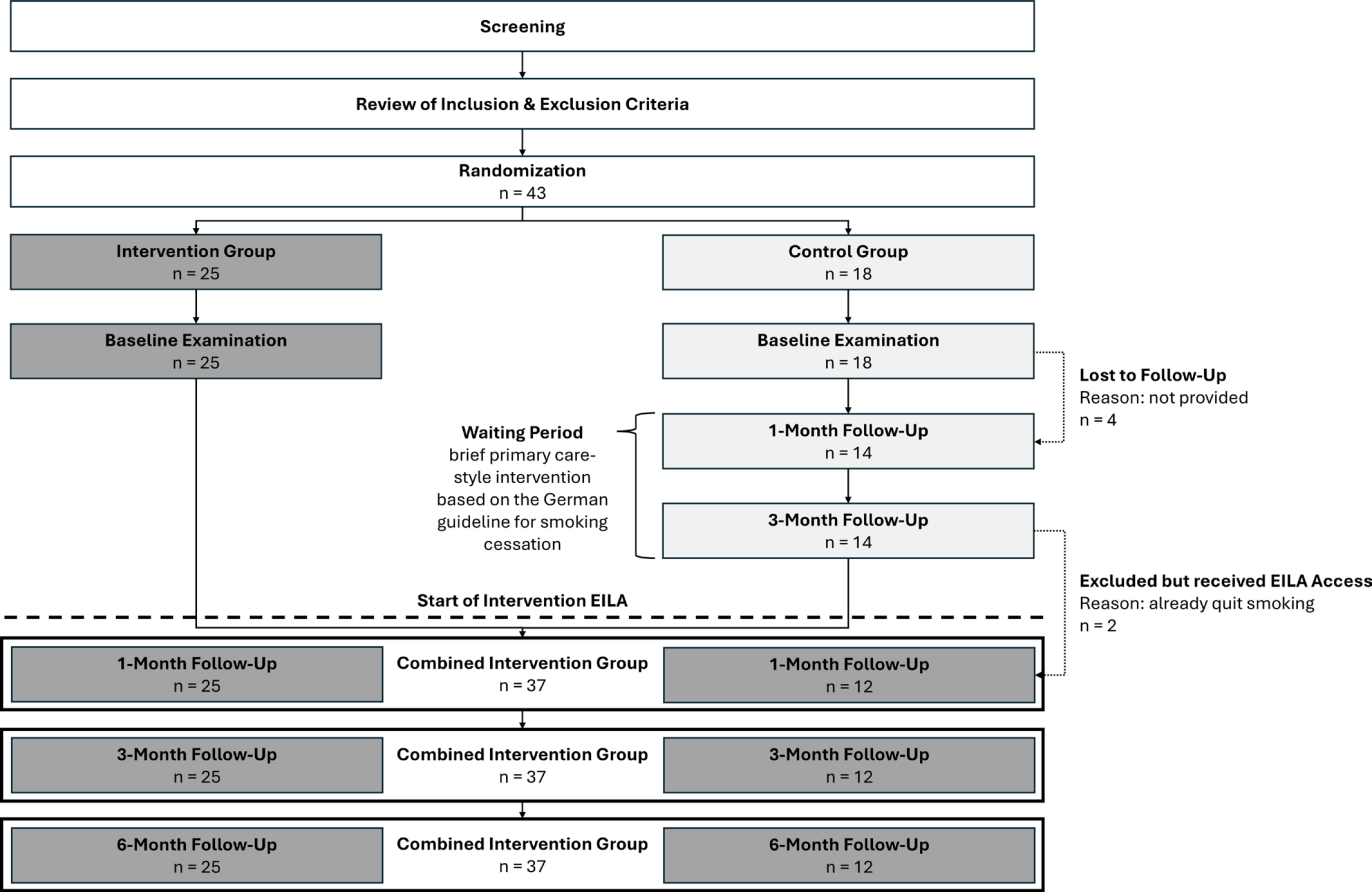
Flow chart of study design. 25 participants were enrolled in the intervention group and 18 in the control group. In the control group, four participants were lost to follow-up and two quit smoking before the end of the waiting period. The remaining 12 participants in the control group subsequently crossed over to the intervention arm, forming the combined intervention group together with those originally assigned to the intervention arm.

This single-centre, randomised controlled trial was conducted at the University Hospital Schleswig-Holstein Lübeck from May 2022 to June 2023. A total of 43 participants met the inclusion criteria: (i) age over 18, (ii) a minimum daily consumption of 10 cigarettes or a Fagerström score ≥3, (iii) ownership of a compatible smartphone, (iv) motivation to quit smoking and (v) no addiction to other psychoactive substances. Although not strictly an inclusion criterion, all recruited hospital workers were employed in the shift work system. Shift work was defined according to the Collective Wage Agreement for the Public Sector in Germany, requiring regular variations in daily start times of at least 2 hours, with identical start times allowed for up to 1 month. The total shift coverage had to span at least 13 hours. Participants were randomly assigned, using sealed envelopes, to either an intervention group (n=25) receiving the EILA application or a control group (n=18) undergoing a brief primary care-style intervention based on the German guideline for smoking cessation.^18^ Subsequently, these two groups were merged into a single combined intervention group as part of a crossover study design (n=37), as detailed in the following section. Follow-up data for the intervention group were collected at baseline and at 1, 3 and 6 months after the start of the intervention. For the control group, follow-up was conducted at baseline, 1 month and 3 months during the waiting period. Afterwards, all participants of the control group gained access to the EILA app, and their 3 month follow-up measurements at the end of the control arm were treated as their new baseline. Similar to the intervention group, they underwent follow-up assessments at 1, 3, and 6 months after starting the intervention, but only the results of participants who continued smoking after completion of the control arm were considered for later analysis and combined with those of the intervention group to form the combined intervention group. Patients who missed follow-up appointments were classified as lost to follow-up, with no subsequent reasons provided for their absence (see figure 1). The sample size was limited by voluntary participation after promotion via company internal email, presentations, flyers, and posters.

### Votum of the ethics committee and registration

This study was reviewed and approved by the Ethics Commission of the University of Lübeck, receiving a positive vote before participant recruitment (reference number 21–344). This approval confirms that the research adheres to ethical standards, ensuring the protection of participants’ rights, dignity and well-being. All participants provided informed consent before enrollment in the study. The study was registered at the German Register of Clinical Studies (DRKS00028466).^19^

### Intervention: the EILA application

The EILA application utilises artificial intelligence to offer individualised smoking cessation support based on users’ unique habits and behaviours.^17^ Initially, the app monitors smoking patterns during a 3 week observation phase, functioning as a digital smoking diary. Participants record smoking events and related contextual information, such as time, emotional state and situation. The app then transitions to an intervention phase, providing personalised behavioural recommendations, feedback and suggestions based on the participant’s input, including alternative strategies and behavioural interventions to manage cravings and reduce the likelihood of smoking.^20^ To maintain user motivation, the application provides real-time displays of health benefits, including days of life gained, financial savings from reduced smoking and smoke-free days. This digital intervention was compared with a single cessation support session designed to reflect a primary care visit, in accordance with the German S3 Guideline ‘Smoking and Tobacco Dependence: Screening, Diagnosis, and Treatment’.^18^

### Smoking status verification: cotinine test

Smoking status was confirmed through cotinine testing using a point-of-care SureStep test ‘COT 100 One Step Cotinine Test Device (Urine)’ that detects urinary cotinine, a metabolite of nicotine, at a threshold of 100 ng/mL, with a specificity of 91% (95% CI 84 to 96%) and a sensitivity of 92% (95% CI 86 to 95%).^21 22^ A negative cotinine test result indicated successful smoking cessation.

### Nicotine dependence: Fagerström Test for Nicotine Dependence

The level of nicotine dependence was quantified using the Fagerström Test for Nicotine Dependence (FTND), a six-item questionnaire that quantifies the severity of smoking dependence. Scores range from 0 to 10, with higher scores indicating greater dependence.^23 24^ This metric enabled a direct comparison of nicotine dependence between baseline and follow-up assessments.

### Desire to smoke assessment: Visual Analogue Scale

Participants rated their current craving to smoke using a Visual Analogue Scale (VAS) specifically designed for this study. The scale ranged from 0 (‘no desire to smoke’) to 10 (‘an extreme desire to smoke’), with participants indicating their craving intensity. This quantitative measure allowed for the assessment of changes in craving intensity in response to the intervention.

### Quality of life measurement: EQ-5D-5L

Health-related quality of life was assessed using the EQ-5D-5L (EQ-5D).^25 26^ This standardised measure includes five dimensions: mobility, self-care, usual activities, pain/discomfort and anxiety/depression. Each was rated on a five-point scale. Additionally, participants rated their overall health on a VAS from 0 (worst health imaginable) to 100 (best health imaginable). This instrument provided a comprehensive measure of quality-of-life changes over the study period and is recognised as a well-established screening tool.

### Depression screening: Patient Health Questionnaire-9

Participants’ mental health was evaluated using the Patient Health Questionnaire-9 (PHQ-9), a widely recognised screening tool for depressive symptoms based on the DSM-5 criteria.^27 28^ Each participant self-reported symptoms on a 9-item scale, with scores indicating the severity of depressive symptoms. Scores <10 were considered to indicate no significant depressive symptoms, while higher scores suggested mild to severe symptoms.

### Assessment of cardiovascular risk: PROCAM score

Cardiovascular risk was evaluated using the PROCAM Score, a validated risk index score developed by the University of Münster.^29^ This index calculates the 10 year risk of a coronary event based on cardiovascular factors such as age, LDL and HDL cholesterol, triglyceride levels, diabetes status, systolic blood pressure and smoking habits. Each participant’s PROCAM score was recorded at baseline and at follow-up assessments to observe changes in cardiovascular risk.

### Statistical analysis

Data analyses were conducted using R.^30^ Power analysis was conducted using estimates for behavioural therapy-based mobile applications.^31^ Based on these results, Cohen’s d was conservatively set at 0.8. Then, the sample size for an α=0.05, and a power of 0.8 was derived from parametric tests and estimated at n=52. Descriptive statistics, including means and SD for continuous variables and scores, were calculated, likewise percentages for categorical variables. Due to the small cohort size, non-parametric tests were used. Baseline characteristics of the study population were compared using the χ^2^ test for categorical variables and the Wilcoxon rank-sum test for ordinal and continuous variables. The Wilcoxon signed-rank test was used on repeated measures. The baseline measurements were set as the reference values. To estimate smoking cessation rates, the relative risk and respective 95% CIs were calculated for each follow-up. All tests were two-tailed. A p-value less than 0.05 was considered statistically significant.

## Results

### Participant characteristics

The study included n=43 participants (n=26 females, n=17 males) with a mean age of 43.3±12.1 years, recruited from the University Hospital Schleswig-Holstein, all of whom were shift workers. Full demographic data are presented in table 1.

**Table 1:** Three groups were defined: the control group, the intervention group and the combined intervention group, which included participants from the intervention group and those participants from the control arm who continued smoking and subsequently crossed over to receive the intervention

	Control group	Intervention group	Combined intervention group	P
N	18	25	37	
Sex				0.78
Female	10 (55.6%)	16 (64.0%)	22 (59.5%)	
Male	8 (44.4%)	9 (36.0%)	15 (40.5%)	
Age (years)				0.54
Mean (SD)	46.01 (14.14)	41.33 (10.55)	43.82 (11.55)	
Weight (kg)				0.56
Mean (SD)	77.72 (17.16)	82.280 (19.86)	80.84 (19.08)	
BMI (kg/m^2^)				0.32
N-Miss	1	0	1	
Mean (SD)	25.23 (4.10)	27.576 (5.78)	26.72 (5.37)	
Pack-years (years)				0.49
Mean (SD)	17.89 (15.70)	26.25 (39.09)	23.57 (32.94)	
FTND (pt)				0.59
N-Miss	0	0	3	
Mean (SD)	4.11 (1.81)	4.88 (1.59)	4.41 (1.93)	
VAS Smoking Desire				0.24
N-Miss	1	0	2	
Mean (SD)	1.47 (2.29)	2.92 (2.82)	2.37 (2.66)	

P values represent comparisons of baseline characteristics between the control group and the intervention group.

BMI, body mass index; FTND, Fagerström Test for Nicotine Dependence; VAS, Visual Analogue Scale.

### Smoking status and cessation rates

**Figure 2:**
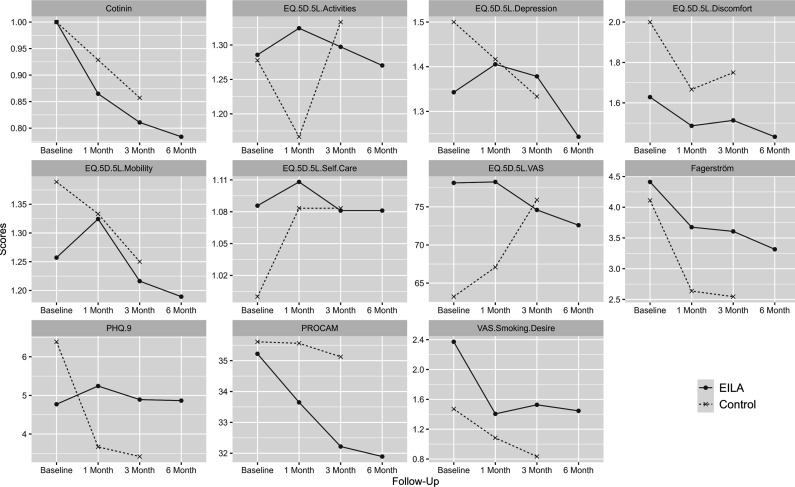
Development of the measurements for the follow-ups. PHQ-9, Patient Health Questionnaire-9; VAS, Visual Analogue Scale.

In the combined intervention group (n=37), the percentage of participants who discontinued smoking at the 1 month follow-up was 13.51% and increased to 18.91% by the 3 month follow-up and 21.62% by the 6 month follow-up. In contrast, in the control group (n=14), the share of participants who quit smoking rose from 7.14% at the first follow-up to 14.28% at the 3 month follow-up (see figure 2). The relative risk for continuing smoking was 0.93 (95% CI 0.77 to 1.13) at the 1 month follow-up and 0.94 (95% CI 0.73 to 1.23) at the 3 month follow-up and 0.91 (95% CI 0.7 to 1.2) at the 6 month follow-up when the cessation rates in the combined intervention group at 6 months were compared with the 3 month follow-up of the control group. These outcomes indicate an effect of the intervention; however, statistical significance was not observed.

### Nicotine dependence

The combined intervention group exhibited a reduction in FTND scores. The FTND scores decreased over time from 4.41±1.93 (n=34) at baseline to 3.68±2.62 (n=34, p=0.028) at 1 month follow-up, to 3.61±2.62 (n=28, p=0.009) at 3 month follow-up, and to 3.32±2.69 (n=19, p=0.014) at 6 month follow-up, highlighting the potential of the application to attenuate nicotine dependence and cravings. However, there was also a decrease in FTND scores in the control group from 4.11±1.81 (n=18) at baseline to 2.64±2.16 (n=11, p=0.03) at 1 month follow-up to 2.55±2.38 (n=11, p=0.039) at 3 month follow-up (see figure 2).

### Desire to smoke

Scoring on the VAS for the desire to smoke decreased for both groups. However, statistical significance was only observed for the combined intervention group with a change from baseline 2.37±2.66 (n=35) to 1.53±1.98 (n=35, p=0.034) at 3 month follow-up and 1.45±1.89 (n=35, p=0.013) at 6 month follow-up (see figure 2).

### Quality of life

EQ-5D scores showed various dynamics. All item scores decreased in the combined intervention group by the 6 month follow-up. Significant changes were only observed for the depression dimension, with 1.34±0.68 at baseline to 1.24±0.72 (n=35, p=0.02) at 6 month follow-up (see figure 2). In comparison to the control group, the items related to self-care, activity, and the scores on the VAS increased at the 3 month follow-up, but overall, no significant changes were observed.

### Depression screening

PHQ-9 scores across both groups remained below 10, indicating low depressive symptoms throughout the study period. The combined intervention group showed no significant changes. A slight decrease was detected in the control group. The score decreased from 6.39±5.72 (n=18) at baseline to 3.42±3.18 (n=12, p=0.037) at 3 month follow-up (see figure 2).

### Assessment of cardiovascular risk

The PROCAM scores showed a decline in the combined intervention group, from a baseline mean of 35.22±12.47 (n=36) to 33.65±14.69 (n=36, p=0.35) at the 1 month follow-up, to 32.22±13.4 (n=36, p=0.01) at the 3 month follow-up and to 31.9±13.24 (n=36, p=0.008) at 6 months follow-up. In the control group, scores remained stable with 35.61±11.15 (n=18) at baseline to 35.56±11.47 (n=16, p=0.82) at the 1 month follow-up and 35.13±11.08 (n=16, p=0.42) at the 3 month follow-up (see figure 2).

## Discussion

This randomised controlled trial among hospital workers in a German university hospital evaluated the efficacy in smoking cessation of the EILA application, a digital smoking cessation tool that combines artificial intelligence and behavioural psychology principles. Concerning the primary endpoint, the findings suggest that the EILA application may reduce smoking rates. However, no statistically significant change in the relative risk was observed. Analysis of secondary endpoints revealed a reduction in nicotine dependence and cardiovascular risk while mental health measures remained stable or showed favourable trends. These results highlight the potential of digital health interventions in tobacco control, particularly within high-risk populations such as healthcare workers. Moreover, these findings are mostly coherent with other studies completed in Germany and other countries.^13 32^

The increase in smoking cessation rates underscores the capacity of digital health applications to support behaviour change. The tailored interventions offered by EILA, including personalised notifications, real-time health benefit feedback, and context-specific strategies, likely contributed to these outcomes. Although statistical significance was not achieved, the observed effect may still be clinically meaningful. Regarding the power analysis, statistically significant effects could arguably be demonstrated in a larger cohort. These findings are consistent with existing literature, demonstrating the effectiveness of personalised, artificial intelligence-driven interventions for smoking cessation.^13 32^

The marked reduction in nicotine dependence, as assessed by the FTND and VAS, further highlights the utility of the application. By targeting individual smoking triggers and habits, EILA may offer benefits beyond those of brief interventions. This aligns with previous studies that emphasise the importance of tailoring cessation strategies to individual needs.^8^ However, the control group also showed a decline in nicotine dependence on the FTND. Possible explanations will be presented within the limitations part.

The observed improvements in depression dimensions of the EQ-5D quality of life measure further highlight the holistic benefits of smoking cessation. Such findings emphasise the potential for digital health interventions to enhance both physical and mental well-being. Moreover, it is worth mentioning that the scores for the PHQ-9 decreased in the control group.

Cardiovascular risk reduction, as reflected in the PROCAM score, is a noteworthy finding, particularly given the high prevalence of smoking-related cardiovascular diseases. The combined intervention group’s marked decrease in PROCAM scores indicates that digital tools like EILA have broader health benefits by promoting smoking cessation. These outcomes are in line with studies linking smoking cessation to rapid cardiovascular risk reduction.^33^ The PROCAM score was therefore used as an integrative indicator to illustrate the potential downstream health effects associated with successful cessation rather than as an independent biomarker.

A key strength of this study is its comprehensive evaluation of different dimensions of smoking cessation, including objective measures such as cotinine testing, validated quality-of-life and mental health tools and cardiovascular risk assessment. The use of a wait-list control group allowed for the evaluation of the app’s additional benefits beyond standard brief interventions, providing robust evidence of its efficacy.

The findings of this study have implications for tobacco control policies and practices as smoking rates among healthcare workers remain a concern. Targeted interventions such as EILA could play a crucial role in addressing this public health challenge. The app’s ability to provide tailored, on-demand support makes it suitable for populations with irregular work schedules and high stress levels. Integrating EILA into existing occupational health programmes is cost-effective and could deliver personalised support to healthcare workers in high-stress environments worldwide, potentially reducing smoking prevalence and related morbidity. Reduced sick leave among clinical staff may, in turn, have a direct positive impact on public health by strengthening the workforce during periods of staff shortages.

Moreover, the integration of digital health applications into existing smoking cessation programmes could enhance their accessibility and scalability. The recent approval of similar apps by the German Federal Institute for Drugs and Medical Devices underscores the growing recognition of digital tools as viable components of tobacco addiction treatment.^9^ Policymakers and healthcare providers should consider incorporating such interventions into broader tobacco control strategies.

Further research is needed to explore the long-term efficacy of digital smoking cessation tools like EILA. Studies involving larger, more diverse populations and extended follow-up periods will be critical in establishing their role in tobacco control. Additionally, investigating the combination of such applications with other cessation aids, such as pharmacotherapy and counselling, could provide insights into possible synergies.

Finally, future studies should explore the potential of digital interventions to address disparities in smoking cessation outcomes. For instance, tailoring applications to specific demographic or cultural groups may enhance their accessibility and impact.

### Limitations of the study

The small sample size may limit the generalisability of the findings to broader populations. The target sample size determined by the power analysis was not reached. Therefore, some potentially significant effects may have gone undetected. However, studies investigating healthcare professionals are rare. Recruitment is challenging due to the demands of clinical roles, and high loss to follow-up is common, a situation further compounded in this study as all participants worked in the shift system with irregular schedules. Larger multicentre trials are needed to validate the reported findings.

Furthermore, the sample sizes of the intervention and control groups were imbalanced. Although the sealed envelopes originally ensured equal group allocation, random assignment, and the lower-than-planned total enrolment led to some control group envelopes remaining unused.

Additionally, the short follow-up period does not allow for an assessment of long-term cessation rates or sustained health benefits. Although a 12 month follow-up was planned in the original study protocol, only two participants attended this assessment, and the others withdrew without explanation, underscoring the difficulty of maintaining participation in a study population of hospital shift workers with intensive and irregular work schedules. Future studies should aim to extend follow-up periods to confirm and expand on these findings.

Another limitation is the potential for bias introduced by self-reported measures, such as the VAS for craving intensity. Although cotinine testing provided an objective verification of smoking status, self-reported data on other outcomes may have been influenced by social desirability or recall bias.^34^ Additionally, the observed effects may be influenced by the Hawthorne effect, whereby hospital workers modify their smoking behaviour in response to awareness of being monitored as study participants, rather than as a direct consequence of the application itself.^35^ Beyond self-reported measures, objective assessment of vascular and pulmonary functional parameters is of considerable interest and was included in the original study protocol. These measures will be evaluated and reported in the context of a separate, dedicated research paper. The present work, in contrast, focuses on self-reported outcomes and smoking cessation rates.

Finally, in the cross-over study design, the control group gained access to the EILA application and became part of the combined intervention group. However, only measurements of participants who continued smoking after the primary style intervention were included in the analysis. Participants in the control that stopped smoking still received access to the EILA application. However, their follow-up results were excluded from the analysis. Given that the primary care-style intervention likely exerted effects early in the study, as evidenced by substantial changes from baseline to the 1 month follow-up in the control group, the additional impact of the EILA application may have been attenuated due to already reduced baseline values. Nonetheless, this approach was adopted to increase the sample size and reduce the risk of overestimating the effects of the intervention.

## Conclusion

The EILA application shows promise as an innovative tool for smoking cessation among hospital workers. By integrating artificial intelligence with behavioural psychology principles, the app targets key determinants of nicotine dependence and smoking behaviour. These findings support the potential of incorporating digital health interventions into comprehensive tobacco control strategies, offering a scalable and accessible approach to addressing one of the leading preventable causes of morbidity and mortality globally.

## Data Availability

Data are available upon reasonable request.

## References

[1] Murray CJL, Aravkin AY, Zheng P (2020). Global burden of 87 risk factors in 204 countries and territories, 1990–2019: a systematic analysis for the Global Burden of Disease Study 2019. The Lancet.

[2] Effertz T, Viarisio V (2015). Die Kosten des Rauchens in Deutschland. Aus der Wissenschaft – für die Politik. Deutsches Krebsforschungszentrum.

[3] Kotz D, Acar Z, Klosterhalfen S (2022). Konsum von Tabak und E-Zigaretten bei Jugendlichen und jungen Erwachsenen über den Zeitraum Juni 2016 bis November. DEBRA Studie.

[4] Nilan K, McKeever TM, McNeill A (2019). Prevalence of tobacco use in healthcare workers: A systematic review and meta-analysis. PLoS One.

[5] Cho Y-M, Kim H-R, Kang M-Y (2019). Fixed night workers and failed smoking cessation. J Occup Med Toxicol.

[6] Abroms LC, Lee Westmaas J, Bontemps-Jones J (2013). A content analysis of popular smartphone apps for smoking cessation. Am J Prev Med.

[7] Bricker JB, Watson NL, Mull KE (2020). Efficacy of Smartphone Applications for Smoking Cessation: A Randomized Clinical Trial. JAMA Intern Med.

[8] Dahlhausen F, Zinner M, Bieske L (2021). Physicians’ Attitudes Toward Prescribable mHealth Apps and Implications for Adoption in Germany: Mixed Methods Study. JMIR Mhealth Uhealth.

[9] Verzeichnis | diga-verzeichnis. https://diga.bfarm.de/de/verzeichnis.

[10] WHO framework convention on tobacco control, World Health Organization (2003). WHO framework convention on tobacco control.

[11] van Amelsvoort LGPM, Jansen NWH, Kant I (2006). Smoking among shift workers: More than a confounding factor. Chronobiol Int.

[12] Rupp A, Hering T, Bubeck A (2023). Leitlinienbasierte digitale Tabakentwöhnung mit PC, Tablet oder Smartphone. Dtsch Med Wochenschr.

[13] Rupp A, Rietzler S, Di Lellis MA (2024). Digital Smoking Cessation With a Comprehensive Guideline-Based App-Results of a Nationwide, Multicentric, Parallel, Randomized Controlled Trial in Germany. Nicotine Tob Res.

[14] DRKS - deutsches register klinischer studien. https://drks.de/search/de/trial/DRKS00031140.

[15] Smoke free - rauchen aufhören, information für fachkreise. https://diga.bfarm.de/de/verzeichnis/01909/fachkreise.

[16] NichtraucherHelden-App, Information für Fachkreise. https://diga.bfarm.de/de/verzeichnis/01085/fachkreise.

[17] Eila-Studie Eila studie. https://eilastudie.de/.

[18] S3-Leitlinie - Rauchen und Tabakabhängigkeit: Screening, Diagnostik und Behandlung (2021). AWMF Arbeitsgemeinschaft der Wissenschaftlichen Medizinischen Fachgesellschaften eV.

[19] DRKS - deutsches register klinischer studien. https://drks.de/search/de/trial/DRKS00028466.

[20] Gonul S, Namli T, Huisman S (2019). An expandable approach for design and personalization of digital, just-in-time adaptive interventions. J Am Med Inform Assoc.

[21] SureStepTM urine drug test cassette. https://www.toxicology.abbott/au/en/screening-devices/surestep-urine-drug-cassette.html.

[22] Yeh E, Levasseur G, Kaiserman MJ (2011). Evaluation of urinary cotinine immunoassay test strips used to assess smoking status. Nicotine Tob Res.

[23] Pomerleau CS, Carton SM, Lutzke ML (1994). Reliability of the Fagerstrom Tolerance Questionnaire and the Fagerstrom Test for Nicotine Dependence. Addict Behav.

[24] Sharma MK, Suman LN, Srivastava K (2021). Psychometric properties of Fagerstrom Test of Nicotine Dependence: A systematic review. Ind Psychiatry J.

[25] Herdman M, Gudex C, Lloyd A (2011). Development and preliminary testing of the new five-level version of EQ-5D (EQ-5D-5L). Qual Life Res.

[26] Feng Y-S, Kohlmann T, Janssen MF (2021). Psychometric properties of the EQ-5D-5L: a systematic review of the literature. Qual Life Res.

[27] Kroenke K, Spitzer RL, Williams JB (2001). The PHQ-9: validity of a brief depression severity measure. J Gen Intern Med.

[28] Levis B, Benedetti A, Thombs BD (2019). Accuracy of Patient Health Questionnaire-9 (PHQ-9) for screening to detect major depression: individual participant data meta-analysis. BMJ.

[29] Assmann G, Cullen P, Schulte H (2002). Simple scoring scheme for calculating the risk of acute coronary events based on the 10-year follow-up of the prospective cardiovascular Münster (PROCAM) study. Circulation.

[30] R Core Team R: a language and environment for statistical computing. https://www.R-project.org.

[31] Pallejà-Millán M, Rey-Reñones C, Barrera Uriarte ML (2020). Evaluation of the Tobbstop Mobile App for Smoking Cessation: Cluster Randomized Controlled Clinical Trial. JMIR Mhealth Uhealth.

[32] Bricker JB, Mull KE, Kientz JA (2014). Randomized, controlled pilot trial of a smartphone app for smoking cessation using acceptance and commitment therapy. Drug Alcohol Depend.

[33] Parmar MP, Kaur M, Bhavanam S (2023). A Systematic Review of the Effects of Smoking on the Cardiovascular System and General Health. Cureus.

[34] Vesely S, Klöckner CA (2020). Social Desirability in Environmental Psychology Research: Three Meta-Analyses. Front Psychol.

[35] Berkhout C, Berbra O, Favre J (2022). Defining and evaluating the Hawthorne effect in primary care, a systematic review and meta-analysis. Front Med (Lausanne).

